# Residential neighbourhood greenspace is associated with reduced risk of incident diabetes in older people: a prospective cohort study

**DOI:** 10.1186/s12889-016-3833-z

**Published:** 2016-11-18

**Authors:** Alice M. Dalton, Andrew P. Jones, Stephen J. Sharp, Andrew J. M. Cooper, Simon Griffin, Nicholas J. Wareham

**Affiliations:** 1 Norwich Medical School, University of East Anglia, Norwich Research Park, Norwich, NR4 7TJ UK; 2 Medical Research Council Epidemiology Unit, University of Cambridge, Cambridge, UK

**Keywords:** Incident diabetes, Physical activity, Greenspace exposure, Older adults

## Abstract

**Background:**

Three cross sectional studies suggest that neighbourhood greenspace may protect against incident diabetes. This study uses data from a longitudinal study with a large sample size to investigate the association between greenspace and the occurrence of incident diabetes over time.

**Methods:**

Data was from the European Prospective Investigation of Cancer Norfolk, UK, cohort, recruitment 1993–2007 (*N* = 23,865). Neighbourhoods were defined as 800 m circular buffers around participants’ home locations, according to their home postcode (zip code). Greenspace exposure was defined as the percentage of the home neighbourhood that was woodland, grassland, arable land, mountain, heath and bog, according to the UK Land Cover Map. Cox proportional hazards regression examined the association between neighbourhood greenspace exposure and incident diabetes. The population attributable fraction assessed the proportion of diabetes cases attributable to exposure to least green neighbourhoods. Mediation analysis assessed if physical activity explained associations between greenspace and diabetes. Interaction analysis was used to test for the modifying effect of rurality and socio-economic status on the relationship between greenspace and diabetes. Models were adjusted for known and hypothesised confounders.

**Results:**

The mean age of participants was 59 years at baseline and 55.1% were female. The mean follow-up time was 11.3 years. Individuals living in the greenest neighbourhood quartile had a 19% lower relative hazard of developing diabetes (HR 0.81; 95% CI 0.67, 0.99; *p* = 0.035; linear trend *p* = 0.010). The hazard ratio remained similar (HR 0.81; 95% CI 0.65, 0.99; *p* = 0.042) after adjusting for age, sex, BMI, whether a parent had been diagnosed with diabetes and socio-economic status at the individual and neighbourhood level. A HR of 0.97 was attributed to the pathway through physical activity in a fully adjusted model, although this was non-significant (95% CI 0.88, 1.08; *p* = 0.603). The incidence of diabetes in the least green neighbourhoods (with 20% greenspace on average) would fall by 10.7% (95% CI −2.1%, 25.2%; *p* = 0.106) if they were as green as the average neighbourhood observed across the whole cohort (59% greenspace on average). There were no significant interactions between rurality or socio-economic status and level of greenspace.

**Conclusions:**

Greener home neighbourhoods may protect against risk of diabetes in older adults, although this study does not support a mediation role for physical activity. Causal mechanisms underlying the associations require further investigation.

**Electronic supplementary material:**

The online version of this article (doi:10.1186/s12889-016-3833-z) contains supplementary material, which is available to authorized users.

## Background

In 2015, 8.8% of adults worldwide were living with diabetes mellitus, at a healthcare cost of over US$673 billion, whilst 5 million people died from the disease [1]. The prevalence of type 2 diabetes is increasing at a fast rate and is anticipated to rise by 55% by 2035 [2]. Lifestyle plays a major role as a risk for type 2 diabetes, with modifiable risk factors such as physical inactivity, unhealthy eating and obesity influencing the development of the disease [3–5]. Wider environmental factors help to determine these behaviours, and should be the target of interventions to reduce disease incidence due to their potential population-level impact [6].

Several studies have found a link between access to greenspace and better physical and mental health [7, 8]. The association of greenspace with type 2 diabetes has been previously examined in three studies, all of which suggested that the likelihood of having diabetes was lower in people living in the greenest areas [9–11]. All three studies calculated the amount of greenspace within 1 km and/or 3 km radii of participant’s home locations, using mapped land use data, and used regression analyses to test the association with type 2 diabetes. However, these cross-sectional studies only looked at prevalent diabetes in participants at a single time point, and one of the three relied on self-reported diabetes diagnosis [9], so the ability to attribute causality to observed associations is limited. In addition, potential causal pathways were not tested, limiting understanding of mechanisms that may explain the relationship between greenspace and diabetes.

Lachowycz and Jones discussed potential causal pathways to explain the relationship between greenspace exposure and disease outcome in their proposed socio-ecological framework [12]. Potential explanatory mechanisms include the physiological and psychological benefits of seeing greenspace [13], the health benefits from the immunoregulatory effects of exposure to microorganisms found in natural environments [14], the role of greenspace in creating a sense of attachment to place and community [15], urban greenspace and its mitigating of air pollution, noise, and the urban heat island effect [16, 17], and its function as a venue for physical activity [18]. Physical activity may be partly determined by the natural and built environment [19], such as greenspace availability [8], although evidence from previous studies has been inconsistent [7]. Physical activity is also a potential mediator of the relationship between exposure to greenspace and diabetes incidence [12], yet previous studies have not tested this [9, 10].

In this study, our primary objective was to use longitudinal data to explore the association between neighbourhood greenspace and incident diabetes in a large population, using a robust, multi-source ascertainment of incident diabetes over follow-up and a detailed objective measure of greenspace exposure in the home neighbourhood. As a secondary objective, we tested physical activity as a potential mediator in this relationship.

## Methods

### Aim, study design and setting

This study uses the European Prospective Investigation of Cancer (EPIC) Norfolk cohort study in the UK, in which data on a wide range of health and lifestyle factors have been obtained over a follow-up period of over two decades [20]. The baseline survey for EPIC-Norfolk was conducted between 1993 and 1997, recruiting 25,639 residents of the region of East Anglia attending 35 general practice surgeries situated in the county of Norfolk [21]. This study uses data from the baseline survey as well as data collected at subsequent follow-up stages up to 2007, the most recent date for which complete diabetes ascertainment was available. The variables used for this analysis are listed in Table 1, with information about the type of measurement used, survey phase and date collected.

**Table 1:** Variables used from the EPIC study measures, with type of measurement and date collected

Variable	Measurement	Survey phase	Date collected
Incident diabetes	Survey, GP records, hospital data	18 month follow-up Health Check 2 Ten year follow up	1994–1998 1996–2000 2003–2007
Home postcode (residential location)	Survey (Health Follow-up 1 Questionnaire)	18 month follow-up	1994–1998
Physical activity	Survey (Health and Lifestyle Questionnaire)	Health Check 1 (baseline)	1993–1997
Height and weight	Physical examination by trained staff	Health Check 1 (baseline)	1993–1997
Demographics, lifestyle and health	Survey (Health and Lifestyle Questionnaire)	Health Check 1 (baseline)	1993–1997

### Diabetes case ascertainment and verification

Incident type 2 diabetes cases were ascertained using multiple data sources, including self-report of doctor-diagnosed diabetes from the second health check or follow-up health and lifestyle questionnaires, self-report of diabetes-specific medication in either of the two follow-up questionnaires or medication brought to the follow-up health check (as described in detail elsewhere [20]). If evidence was available from fewer than two of these independent sources, information was verified through record linkage with the general practice diabetes register, local hospital diabetes register, hospital admissions data and Office of National Statistics mortality data with coding for diabetes. Follow-up began at the date of recruitment to the survey and ended at either 31 December 2007, date of diabetes diagnosis or date of death if the participant died before this date.

### Exposure to neighbourhood greenspace

The main explanatory variable was the percentage of land cover in the participant’s home neighbourhood that was classified as greenspace. The ArcGIS 10.1 geographic information system (GIS) software [22], was used to delineate neighbourhood boundaries around participants’ home locations defined according to their home postcode (zip code), reported in the Health Follow-up 1 Questionnaire. For those who had moved address during the follow-up, the neighbourhood greenness was also assessed for the second address. As no information on the exact date of moves was available, we measured the average neighbourhood greenness across the two addresses for these participants. Every postcode was geo-located using the UK Ordnance Survey Code-Point® database [23], which provides a set of coordinates depicting the average latitude and longitude of all mail delivery locations within each postcode. Each postcode contains 15 addresses, on average.

Estimates of neighbourhood greenness were generated using data from the Centre for Ecology and Hydrology Land Cover Map of the UK (2007) [24], which is derived from satellite images and digital cartography. It records the dominant land use type, based on a 23 class typology, in 25 m by 25 m size grid cells with greenspace being classified as cells that contain broadleaved and coniferous woodland, arable land, improved grassland, semi-natural grassland, mountain, heath and bog for the purposes of this analysis. Each participant’s neighbourhood exposure was computed by overlaying the summed, mapped greenspace with each participant’s neighbourhood boundary in the GIS software.

Neighbourhoods are typically defined as the area within 800 m (approximating equivalent to a ten minute walk) of a home location [25]. However, recent research from studies employing global positioning systems to track movement suggests that this may be overly conservative, and that individuals typically travel greater distances to access resources and undertake physical activity [26]. Given that information on actual movement patterns for the participants of EPIC-Norfolk was not available, the sensitivity of findings to neighbourhood definition was examined by employing three neighbourhood measures: 800 m, 3 km and 5 km. To compute each measure, a circular buffer was used to measure the percentage of the area of each circle that was greenspace. To further test for sensitivity, buffers were also defined according to road network distance, measured using Ordnance Survey Meridian 2 road network data [27].

### Covariates and confounders

Demographic, lifestyle, health and anthropometric characteristics, collected using the baseline Health and Lifestyle Questionnaire, were chosen for this analysis based on empirical evidence and theoretical relevance of associations with incident diabetes and greenspace. This included information about age, sex, family history of diabetes and BMI computed from measured height and weight data [28]. The relationship between greenspace and diabetes might be confounded by socio-economic status (SES) [29]. Therefore, the analysis was adjusted for SES at both the individual and neighbourhood level. Employment derived social class was used at the individual level, classed as manual (skilled manual, semi-skilled, unskilled) and non-manual (professional, managerial and technical, skilled non-manual). At the neighbourhood level (Census enumeration district), we used the Townsend Index, a measure of relative deprivation based on information about employment, care ownership, home ownership and household overcrowding from the UK Census [30]. Ethnicity has been found to be associated with diabetes risk [31], however it was not included in this analysis as 99.6% of the sample (*N* = 23,688) were white.

### Mediation

Physical activity has been previously shown to be directly associated with diabetes incidence within the EPIC-Norfolk cohort, whereby recreational and overall physical activity have been found to reduce the risk of developing type 2 diabetes [32]. To investigate the potential mediating influence of physical activity, we derived a summary ordered categorical index of physical activity which has previously been shown to be valid and repeatable [33, 34]. The original questionnaire [35] asked the participant to report frequency and duration of time spent walking or cycling (for work, shopping and leisure) or engaged in other physical activity (such as swimming, jogging and other activities), along with, for those in employment, the amount of physical activity involved in their work (sedentary, standard, physical or heavy manual work). The first measure used was an overall physical activity indicator, using a combined work and leisure physical activity variable, classifying the participant into one of four activity levels (active, moderately active, moderately inactive or inactive), previously derived from a validated measure [34]. Individuals were assigned to one of these levels based on their overall physical activity level, determined by their responses to two questions asking about their activity in the last 12 months: physical activity at work (sedentary, standing, physical and manual); and time spent cycling and doing other physical exercise each week, categorised into four levels (0, 0–3.5, 3.5–7, >7 h). Those not reporting occupational physical activity were assigned to the sedentary group for occupation.

We also analysed a second variable of a four level measure of engagement in cycling, and other sports less likely to be carried out in greenspaces. Finally, we analysed the reported number of hours spent walking each week in summer, divided into quartiles, and theorised to be activities most likely to be carried out in greenspaces.

### Data analysis

The direct association between greenspace and incident diabetes was estimated using Cox proportional hazards regression models, with age as the underlying timescale [36]. Entry time was defined for each participant as age at baseline, and exit time as age at diagnosis of diabetes, censoring (participant did not develop diabetes by the end of 2007, were lost to follow-up or withdrew from the study), or death (whichever came first).

Cox models were used to estimate associations between individual/area level factors and hazard of diabetes. Model 1 included only the exposure, quantiles of greenspace. Model 2 was also adjusted for age, sex, BMI, whether a parent had been diagnosed with diabetes, and SES. Kaplan-Meier estimates of the probability of remaining free of diabetes were plotted [37]. The population attributable fraction (PAF) and 95% CIs were calculated, representing the proportion of incident diabetes cases that would be prevented if neighbourhoods in the lowest quartile of greenspace were greened to the level of those observed across the whole sample. This used the Stata command ‘punafcc’, a method used previously with survival data [38].

The nature, meaning and use of greenspace may differ between urban and rural areas, with green urban areas particularly tending to be accessible and managed. We therefore stratified the regression models by urban-rural status to investigate if rurality moderated the association between greenspace and incident diabetes. We also explored the potential effect modification by SES by stratifying the regression models by individual-level social class (manual/non-manual occupation).

Mediation analysis was performed to determine whether physical activity wholly or partly explained any associations between greenspace and incident diabetes. Standard techniques for carrying out mediation analysis, based on methods developed by Baron and Kenny [39] and Preacher and Hayes [40], cannot be applied to survival data, which are typically non-normally distributed and right censored [41]. We therefore used the method of Lange and colleagues, which is compatible with survival analysis, to estimate the direct and indirect effects between exposure and outcome [42, 43]. Mediation analysis was carried out using R [44]. All other analyses were conducted using Stata version 13 [45].

## Results

### Sample characteristics

Of the 25,639 participants at baseline, we excluded 1087 who did not have a valid postcode that allowed their residential location to be determined. An additional 113 participants who resided more than 20 km from Norfolk by the time of the 18 month follow-up were excluded, as well as 562 who self-reported diabetes in the baseline questionnaire, five with uncertain diabetes status, and seven with no age at either diagnosis or exit from the study. There were no statistically significant differences in participant characteristics between those excluded and those included in the analysis (the final sample), except for age (mean 60.7 in excluded versus 59.1 years, *p* < 0.001) and BMI (27.0 versus 26.6 kg/m^2^, *p* = 0.012). Six participants with incident diabetes but with no known date of diagnosis were given the median date of the final sample. A total of 23,865 participants were included in the analysis with a mean age at baseline of 59.1 years (minimum 39.5 years, maximum 79.1 years) (Table 2). Of the total sample, 1486 people (6.2%) had moved house by the time of the ten-year follow-up. A total of 834 participants developed incident type 2 diabetes by the end of 2007.

**Table 2:** Baseline characteristics of men and women in EPIC Norfolk

Characteristic	Men (*N* = 10722)	Women (*N* = 13143)	All (*N* = 23865)
Incident diabetes, % (n)	4.4 (473)	2.7 (361)	3.5 (834)
Age (years)	59.4 *±* 9.3	58.8 *±* 9.3	59.1 *±* 9.3
BMI (kg/m^2^)	26.5 *±* 3.3	26.2 *±* 4.3	26.3 *±* 3.9
Parent had diabetes	9.6 (1023)	10.5 (1379)	10.1 (2402)
Social class			
Professional	7.8 (801)	1.6 (196)	4.4 (997)
Managerial	38.4 (3936)	28.9 (3607)	33.2 (7543)
Skilled non manual	11.9 (1216)	39.4 (4927)	27.0 (6143)
Skilled manual	25.9 (2657)	6.6 (822)	15.3 (3479)
Semi-skilled	13.2 (1349)	16.8 (2103)	15.2 (3452)
Unskilled	2.8 (289)	6.7 (836)	5.0 (1125)
Townsend Index of deprivation^a^	−2.1 *±* 2.2	−2.0 *±* 2.2	−2.0 *±* 2.2
Overall physical activity			
Inactive	30.6 (3276)	30.2 (3963)	30.3 (7239)
Moderately inactive	24.5 (2631)	32.1 (4216)	28.7 (6847)
Moderately active	23.0 (2367)	22.3 (2933)	22.6 (5400)
Active	21.9 (2347)	15.5 (2031)	18.3 (4378)
Leisure physical activity (hrs per wk cycling/sport)			
0	54.9 (5890)	50.9 (6692)	52.7 (12582)
> 0– < 3.5	26.7 (2864)	33.0 (4340)	30.2 (7204)
> =3.5–< 7	11.3 (1210)	10.6 (1392)	10.9 (2602)
> =7	7.1 (758)	5.5 (719)	6.2 (1477)
Walking in summer (hrs per wk)	10.1 *±* 11.0 (10102)	9.4 *±* 10.1 (12230)	9.7 *±* 10.5 (22332)
Urban/rural location			
Urban	46.7 (5011)	46.9 (6169)	46.9 (11180)
Town and fringe	20.4 (2188)	20.9 (2749)	20.7 (4937)
Village	23.8 (2554)	23.4 (3077)	23.6 (5631)
Hamlet/isolated dwelling	9.0 (969)	8.7 (1148)	8.9 (2117)
Greenspace (percentage < =800 m home)	59.2 *±* 29.6	58.9 *±* 29.6	59.0 *±* 29.6

Results are % (N) or mean ± SD

^a^A standardised index of between −6.7 (relatively affluent) to +7.0 (relatively deprived), where a score of 0 represents an area with overall mean values

### Greenspace and incident diabetes

**Fig. 1:**
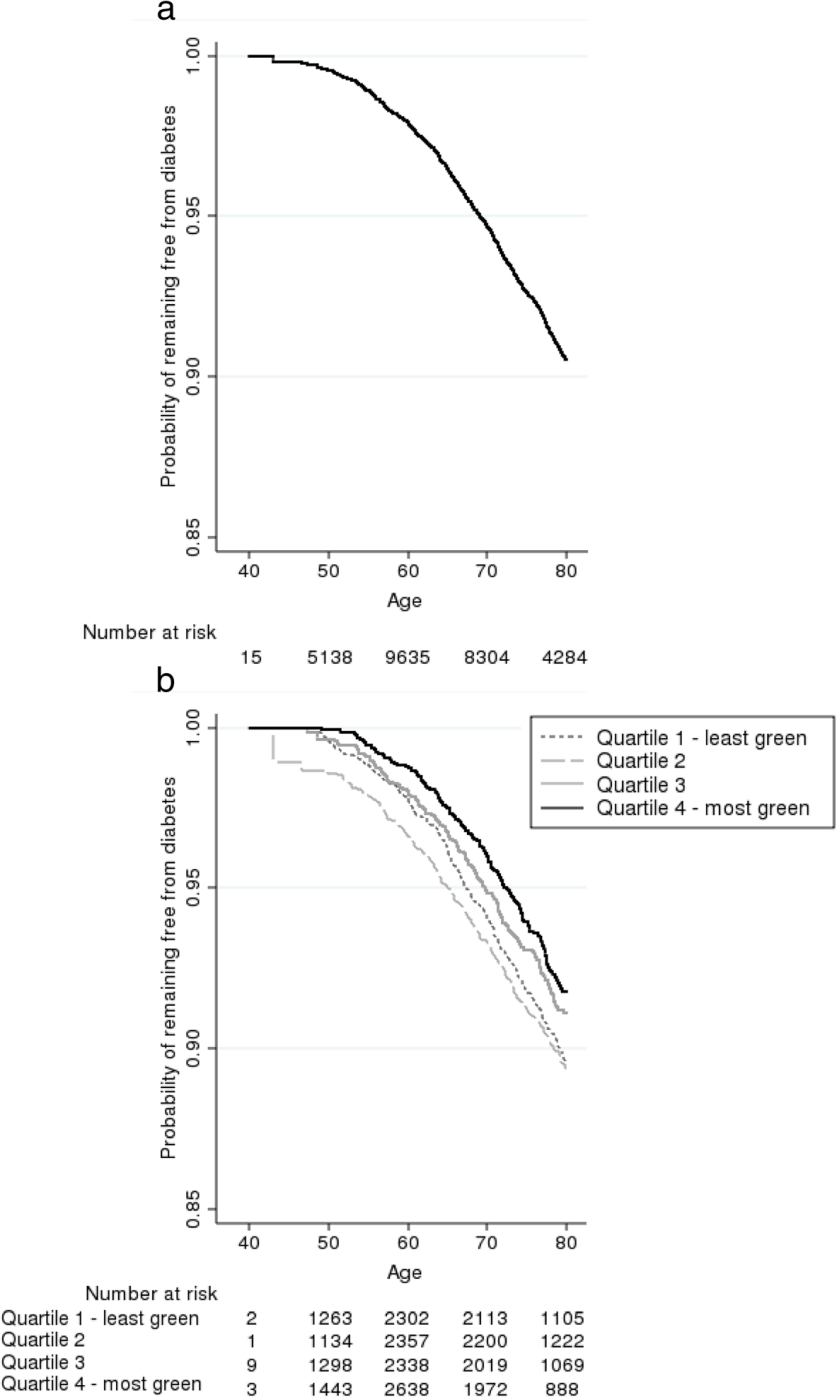
Kaplan-Meier survival curves showing the probability of remaining free of diabetes since baseline. In (**a**) the overall sample and in (**b**) categories based on quartiles of percentage of total land area of participants’ home neighbourhood that is greenspace. Age is used as the underlying timescale. Probabilities only presented up to age 80 due to small numbers of participants older than this

Rates of incident diabetes increased from 7.3 per 10,000 person-years for those aged 40–49 years to 55.2 per 10,000 person-years (1 in 181) for those aged 80–90 years. Figure 1a shows the cumulative survival for the cohort not developing diabetes to be 90.6% at age 80 (data not shown after age 80 due to few observations). There was a significant trend in the survivor functions (i.e., probability of remaining free from diabetes) across the quartiles of greenspace (*p* = 0.010) (Fig. 1b); the probability of remaining free from diabetes amongst those in the greenest areas was 2.4% higher than those in the least green at age 80.

Table 3 presents hazard ratios (HR), confidence intervals (CI) and *p*-values from the Cox models. Before adjustment for confounders, individuals living in the greenest quartile (Q4) had a 19% lower relative hazard of developing diabetes (HR 0.81; 95% CI 0.67, 0.99; *p* = 0.010) compared to those living in the least green quartile (Model 1). The linear trend across quartiles was statistically significant (HR 0.92; 95% CI 0.87, 0.98; *p* = 0.010). The hazard ratio remained similar (HR 0.81; 95% CI 0.65, 0.99; *p* = 0.042) after adjusting for age, sex, BMI, whether a parent had been diagnosed with diabetes and SES (Model 2), with a statistically significant trend across quartiles (HR 0.92; 95% CI 0.86, 0.99; *p* = 0.017).

**Table 3:** Hazard ratios from Cox regression, showing the association between neighbourhood greenspace exposure and incident diabetes

	Model 1 Adjusted for greenspace					Model 2 Adjusted for confounders				
	95% CI					95% CI				
	HR	Lower	Upper	p	p trend	HR	Lower	Upper	p	p trend
Greenspace quartile										
1 (least green, ref)	1.00					1.00				
2	0.96	0.80	1.15	0.674	0.010	0.97	0.80	1.18	0.768	0.017
3	0.81	0.67	0.98	0.031		0.83	0.67	1.02	0.075	
4 (most green)	0.81	0.67	0.99	0.035		0.81	0.65	0.99	0.042	

Age is used as the underlying time scale. *N* = 23865. Model 2 adjusted for confounders of sex, age, BMI, parental diabetes, and SES

*CI* confidence interval

Computation of the population attributable fraction (PAF) suggested that, based on the model before adjustment, incident diabetes in the least green neighbourhoods (quartile 1, 20% greenspace on average) would fall by 11.4% (95% CI −0.5%, 24.8%; *p* = 0.062) should those neighbourhoods be greened to a level observed across the whole sample, which was 59% greenspace coverage of land area on average. After full adjustment, the corresponding estimate decreased slightly to 10.7% and fell just outside of statistical significance (95% CI −2.1%, 25.2%; *p* = 0.106).

### Assessment of mediation and moderation

The direction of association between greenspace and physical activity was positive for overall activity, but in the opposite direction for hours spent walking and in cycling and sports, whereby activity decreased with greenspace. Mediation was therefore only conducted for overall physical activity.

Mediation analysis suggested that, before adjustment, physical activity partially mediated the association between exposure to greenspace in the home neighbourhood and incident diabetes. The HR of incident diabetes was 0.81 for people living in the greenest home neighbourhoods compared to those living in the least green areas. A HR of 0.96 (95% CI 0.88, 1.06; *p* = 0.452) was attributed to the pathway through physical activity. When covariates of age, BMI, sex, parental diabetes and SES were added to the analysis, the HR of 0.97 remained non-statistically significant (95% CI 0.88, 1.08; *p* = 0.603).

Area greenspace and urban/rural status were strongly associated, but stratification by urban-rural status revealed no evidence of moderation of the association between greenspace and incident diabetes by rurality. There was no significant interaction between SES and greenspace exposure in the regression models, therefore we conclude that modification of the relationship between greenspace and incident diabetes by SES is not present.

### Sensitivity analysis

Sensitivity analysis suggested that using road network buffers rather than circular buffers resulted in only a small change to the hazard ratio in regression analysis. In fully adjusted models, the relative hazard of developing diabetes was slightly lower when neighbourhoods were measured according to road buffers, at 22% (HR 0.78; 95% CI 0.64, 0.96; *p* = 0.023). The indirect effect attributable to physical activity remained non-statistically significant (HR 0.97; 95% CI 0.88, 1.07; *p* = 0.601). Greenspace measured using larger buffer sizes was not statistically significantly associated with incident diabetes. See Additional file 1 for the results of the sensitivity analysis.

## Discussion

The findings of this study suggest that exposure to greenspace may be protective against the development of type 2 diabetes. Participants living in the greenest locations had a 19% lower relative risk of developing diabetes at follow-up when compared to those living in the least green areas. After adjustment for potential confounding by age, sex, BMI, parental diabetes, and SES, the risk reduction remained similar and the relationship remained statistically significant. Our study does not support a mediation role for physical activity in the relationship between exposure to greenspace and incident diabetes. Interestingly, two of the measures of physical activity – walking and time spent doing cycling and other sports – were negatively associated with neighbourhood greenspace. This counter-intuitive finding could be due to residual confounding or measurement error, but it is most likely due high levels of utilitarian walking in urban areas, a factor which we were unable to separate out from leisure walking in the data.

This study is in agreement with the findings from the three previous cross-sectional studies exploring the association between greenspace exposure and diabetes, that type 2 diabetes tends to be lower in greener areas, even after accounting for covariates. However, ours is the only study to look at incident diabetes rather than prevalence of the disease. Maas et al. [11] found that prevalence fell from 10/1000 for participants living in areas with 10% greenspace in a 1 km neighbourhood, to 8/1000 for 90% greenspace; and that a 10% increase in greenspace was associated with a 2% lower prevalence in diabetes in both 1 km and 3 km neighbourhoods. Unlike our study, this did not take BMI and family history of diabetes into account. Our participants lived in greener environments, in an average of 59% greenspace compared to 42.4% in their study. Interestingly, Bodicoat et al. [10] did not find a significant relationship for a 800 m neighbourhood, but found a 3 km neighbourhood to indicate the odds ratio for diabetes of 0.67 in the highest compared with lowest greenspace quartile after adjustment for ethnicity, age, sex, area deprivation and urban/rural location. Their study participants lived in areas with an average of 57% neighbourhood greenspace coverage. Astell-Burt et al. [9] found no relationship for people living in the greenest (>80%) areas, but they found that the odds of having diabetes was 0.90 for those living in 41–60% greenspace in their fully adjusted model. Conversely, we found our greenest quartile, 95% greenspace on average, to be significantly associated with elevated diabetes risk.

Greenspace in the home neighbourhood may be potentially used for physical activity. If this is the case, it should be accessible for people to actively use, and promoted for this use. On the other hand, these results suggest that the relationship between greenspace exposure and incident diabetes remains partly unexplained. This may be due to measurement error of exposure and outcome, residual confounding between greenspace and diabetes risk, and the fact that we had an overall measure of physical activity, rather than just that conducted in greenspace. Other explanatory mechanisms in addition to physical activity might explain the causal link, such as the advantages of exposure to nature for immunological regulation [14]. Increasing the amount of greenspace in the home neighbourhood may reduce incident diabetes in older populations, although without knowing the other mechanisms through which the effects are beneficial, we may not be able to tailor interventions to need. For example, if immunological regulation was found to be involved, the provision of places rich in biodiversity may be key. Alternatively, if causal mechanisms are related to stress-associated inflammatory responses, improving views and increasing the aesthetic value of greenspaces may be important.

The research has a number of strengths. The multiple data source used for ascertainment of type 2 diabetes provided a robust classification of disease incidence. A previous study examining the relationship between exposure to greenspace and diabetes used only self-report data [9], which is subject to error [46]. The large sample size and length of follow-up, with a total of 834 incident cases over a period of 16 years in 23,835 older adults, enabled us to estimate associations between greenspace and incident diabetes with a reasonable degree of precision. In addition, we have used a new and robust method of mediation appropriate for use with survival data. Previous analyses have needed to rely on other methods which have been either too crude or restrictive [43]. It is also noted that despite to the historical nature of the postcodes used for this analysis, only 4.2% of participant addresses could not be matched with a geographical location, representing a small proportion of the total sample. This meant we were able to compute home neighbourhood buffers based on the home address of individuals. Finally, we tested different classifications of exposure to greenspace by running the models on different neighbourhood buffer sizes and types.

In terms of limitations, we had no information about use of greenspace amongst our participants. It may be that physical activity in greenspace is more protective for diabetes, rather than physical activity conducted indoors but we are unable to determine this. Indeed, other research has outlined the possibility of additional health and wellbeing benefits associated with activity outdoors, due to greater enjoyment and satisfaction from the interaction with nature [47]. Further the greenspace measure did not represent the area of publicly accessible greenspace in the home neighbourhood, although it is unclear if greenspace needs to be publicly accessible or just visible to have health benefits. Of the participants who had moved by the ten year follow-up, we did not know how long they resided at each address and therefore we assumed they resided at each address for an equal time, although sensitivity analysis suggested that findings were not strongly altered when using only baseline residential address (results not presented). Our physical activity measurement was based on self-reported data which may be subject to error, although validation exercises have shown this measure to be both valid and repeatable [33, 34].

We were not able to assess quality of greenspaces within the neighbourhood, yet some research suggests that more attractive, litter-free environments improve health outcomes [48]. In the absence of such data, we used detailed land cover information with circular buffers to indicate a potential maximum accessible greenspace, testing the sensitivity by using different sized buffers. Other measures of greenspace have been employed in previous research, such as distance to nearest greenspace or number and size of greenspaces around a home location [12], although these are based on various assumptions around greenspace use. In the absence of a clear causal mechanism linking greenspace exposure to diabetes risk we did not test them.

One other limitation was that the study was conducted in Norfolk and may not be representative of other areas. In particular, there was a lack of ethnic heterogeneity in the sample, as over 99% of the participants were white. However, a benefit of this setting was that the sample had high heterogeneity in greenspace exposure and was drawn from a variety of urban and rural locations across the county. Additionally, we did not know the type of incident diabetes, so we were unable to explore if the relationship between greenspace exposure and disease outcome differed between type 1 and type 2 diabetes. However, in a population aged over 40 at baseline, the incidence of type 2 diabetes is expected to be around 8 per 1000 person-years follow-up [49], compared to an expected incidence of type 1 diabetes of around 8 per 100,000 person-years follow-up [50]. As such, almost all new cases were most likely to be type 2.

## Conclusions

In conclusion, greener home neighbourhoods appear to offer protection against the risk of incident diabetes in older people, a relationship that was not associated with overall physical activity. Other potential causal mechanisms should be explored, including the psychological and social benefits of greenspace and its potential for immune regulation.

## Additional file

Additional file 1: Sensitivity testing. Hazard ratios from Cox regression, showing the association between neighbourhood greenspace exposure and incident diabetes, according to different definitions of neighbourhood and exposure. (DOCX 20 kb)

## Abbreviations

BMI: Body mass index
CI: Confidence interval
EPIC-Norfolk: European prospective investigation of cancer Norfolk
GIS: Geographic information system
HR: Hazard ratio
PAF: Population attributable fraction
UK: United Kingdom
