# Supplementary material for: Residential neighbourhood greenspace is associated with reduced risk of incident diabetes in older people: a prospective cohort study
Source: BMC Public Health. 2016 Nov 18;16:1171. doi: 10.1186/s12889-016-3833-z (PMC5116148; doi:10.1186/s12889-016-3833-z)
Supplement: Additional file 1: — Sensitivity testing. Hazard ratios from Cox regression, showing the association between neighbourhood greenspace exposure and incident diabetes, according to different definitions of neighbourhood and exposure. (DOCX 20 kb) [file 12889_2016_3833_MOESM1_ESM.docx]

Additional File 1: Sensitivity testing

Hazard ratios from Cox regression, showing the association between neighbourhood greenspace exposure and incident diabetes, according to different definitions of neighbourhood and exposure

| 800m circular buffer | Model 1  Adjusted for greenspace | | | | |  | Model 2  Adjusted for confounders | | | | |
| --- | --- | --- | --- | --- | --- | --- | --- | --- | --- | --- | --- |
|  |  | 95% CI | |  |  |  |  | 95% CI | |  |  |
|  | HR | Lower | Upper | p | p trend |  | HR | Lower | Upper | p | p trend |
| Greenspace quartile  1 (least green, ref) | 1.00 |  |  |  | 0.010 |  | 1.00 |  |  |  | 0.017 |
| 2 | 0.96 | 0.80 | 1.15 | 0.674 |  |  | 0.97 | 0.80 | 1.18 | 0.768 |  |
| 3 | 0.81 | 0.67 | 0.98 | 0.031 |  |  | 0.83 | 0.67 | 1.02 | 0.075 |  |
| 4 (most green) | 0.81 | 0.67 | 0.99 | 0.035 |  |  | 0.81 | 0.65 | 0.99 | 0.042 |  |
|  |  |  |  |  |  |  |  |  |  |  |  |
| Indirect effect via physical activity | HR 0.96 (95% CI 0.88, 1.06; p=0.452) | | | | |  | HR 0.97 (95% CI 0.88, 1.08; p=0.603) | | | | |

| 800m road buffer | Model 1  Adjusted for greenspace | | | | |  | Model 2  Adjusted for confounders | | | | |
| --- | --- | --- | --- | --- | --- | --- | --- | --- | --- | --- | --- |
|  |  | 95% CI | |  |  |  |  | 95% CI | |  |  |
|  | HR | Lower | Upper | p | p trend |  | HR | Lower | Upper | p | p trend |
| Greenspace quartile  1 (least green, ref) | 1.00 |  |  |  | 0.010 |  | 1.00 |  |  |  | 0.023 |
| 2 | 0.93 | 0.78 | 1.12 | 0.468 |  |  | 0.91 | 0.75 | 1.10 | 0.344 |  |
| 3 | 0.87 | 0.72 | 1.06 | 0.163 |  |  | 0.89 | 0.73 | 1.09 | 0.255 |  |
| 4 (most green) | 0.78 | 0.64 | 0.95 | 0.013 |  |  | 0.78 | 0.64 | 0.96 | 0.019 |  |
|  |  |  |  |  |  |  |  |  |  |  |  |
| Indirect effect via physical activity | HR 0.96 (95% CI 0.88, 1.06; p=0.449) | | | | |  | HR 0.97 (95% CI 0.88, 1.07; p=0.601) | | | | |

| 3km circular buffer | Model 1  Adjusted for greenspace | | | | |  | Model 2  Adjusted for confounders | | | | |
| --- | --- | --- | --- | --- | --- | --- | --- | --- | --- | --- | --- |
|  |  | 95% CI | |  |  |  |  | 95% CI | |  |  |
|  | HR | Lower | Upper | p | p trend |  | HR | Lower | Upper | p | p trend |
| Greenspace quartile  1 (least green, ref) | 1.00 |  |  |  | 0.227 |  | 1.00 |  |  |  | 0.229 |
| 2 | 0.80 | 0.66 | 0.96 | 0.018 |  |  | 0.85 | 0.69 | 1.04 | 0.109 |  |
| 3 | 0.84 | 0.70 | 1.02 | 0.072 |  |  | 0.82 | 0.67 | 1.00 | 0.053 |  |
| 4 (most green) | 0.87 | 0.72 | 1.05 | 0.156 |  |  | 0.88 | 0.72 | 1.08 | 0.226 |  |

| 3km road buffer | Model 1  Adjusted for greenspace | | | | |  | Model 2  Adjusted for confounders | | | | |
| --- | --- | --- | --- | --- | --- | --- | --- | --- | --- | --- | --- |
|  |  | 95% CI | |  |  |  |  | 95% CI | |  |  |
|  | HR | Lower | Upper | p | p trend |  | HR | Lower | Upper | p | p trend |
| Greenspace quartile  1 (least green, ref) | 1.00 |  |  |  | 0.236 |  | 1.00 |  |  |  | 0.190 |
| 2 | 0.99 | 0.82 | 1.19 | 0.875 |  |  | 1.02 | 0.84 | 1.25 | 0.844 |  |
| 3 | 0.85 | 0.70 | 1.03 | 0.093 |  |  | 0.85 | 0.69 | 1.05 | 0.130 |  |
| 4 (most green) | 0.93 | 0.77 | 1.13 | 0.476 |  |  | 0.92 | 0.75 | 1.14 | 0.446 |  |

| 5km circular buffer | Model 1  Adjusted for greenspace | | | | |  | Model 2  Adjusted for confounders | | | | |
| --- | --- | --- | --- | --- | --- | --- | --- | --- | --- | --- | --- |
|  |  | 95% CI | |  |  |  |  | 95% CI | |  |  |
|  | HR | Lower | Upper | p | p trend |  | HR | Lower | Upper | p | p trend |
| Greenspace quartile  1 (least green, ref) | 1.00 |  |  |  | 0.285 |  | 1.00 |  |  |  | 0.319 |
| 2 | 0.87 | 0.72 | 1.06 | 0.166 |  |  | 0.97 | 0.79 | 1.19 | 0.736 |  |
| 3 | 0.93 | 0.77 | 1.13 | 0.467 |  |  | 0.94 | 0.77 | 1.15 | 0.543 |  |
| 4 (most green) | 0.88 | 0.72 | 1.06 | 0.180 |  |  | 0.90 | 0.74 | 1.11 | 0.338 |  |

| 5km road buffer | Model 1  Adjusted for greenspace | | | | |  | Model 2  Adjusted for confounders | | | | |
| --- | --- | --- | --- | --- | --- | --- | --- | --- | --- | --- | --- |
|  |  | 95% CI | |  |  |  |  | 95% CI | |  |  |
|  | HR | Lower | Upper | p | p trend |  | HR | Lower | Upper | p | p trend |
| Greenspace quartile  1 (least green, ref) | 1.00 |  |  |  | 0.535 |  | 1.00 |  |  |  | 0.552 |
| 2 | 0.83 | 0.69 | 1.01 | 0.062 |  |  | 0.90 | 0.73 | 1.11 | 0.321 |  |
| 3 | 0.91 | 0.75 | 1.09 | 0.310 |  |  | 0.89 | 0.73 | 1.09 | 0.264 |  |
| 4 (most green) | 0.91 | 0.76 | 1.11 | 0.359 |  |  | 0.94 | 0.76 | 1.15 | 0.539 |  |

*Age is used as the underlying time scale. N=23865. Model 2 adjusted for confounders of sex, age, BMI, parental diabetes and SES. CI: confidence interval.*
